# Prevalence of Smoking Among Spontaneous Pneumothorax Patients and Its Impact on Treatment in Syria Country: A Cross‐Sectional Study

**DOI:** 10.1002/hsr2.70743

**Published:** 2025-04-18

**Authors:** Muhanad Munzer, Nafiza Martini, Mhd Mustafa Albitar, Lilas Channiss, Mohammed Martini, Hussam Al bardan

**Affiliations:** ^1^ Damascus University, Faculty of Medicine Damascus Syrian Arab Republic; ^2^ Stemosis for Scientific Research Damascus Syrian Arab Republic; ^3^ Faculty of Medicine University of Illinois Chicago IL USA; ^4^ Department of Internal Medicine, Memorial Medical Center Southern Illinois University School of Medicine Springfield Illinois USA

**Keywords:** chest, conservative treatment, intercostal drainage, recurrence, smoking, spontaneous pneumothorax, surgery

## Abstract

**Background and Aims:**

Spontaneous Pneumothorax (SP) is a case where air is collected in the pleural space, with smoking recognized as a major risk factor. Despite the global burden of SP, there is limited research on its prevalence and recurrence in Middle Eastern populations, particularly in Syria. This study aims to evaluate the prevalence of smoking among SP patients in Syria and investigate its impact on SP recurrence, providing insights that could guide more effective treatment strategies in regions with high smoking rates.

**Methods:**

A cross‐sectional study was conducted on patients with spontaneous pneumothorax treated at two university hospitals in Syria from January 2016 to January 2021. Data on demographics, smoking habits, treatment types (conservative, intercostal drainage, and surgical), and recurrence were collected. Pearson's Chi‐square test was used to analyze the association between smoking and SP recurrence.

**Results:**

This study included 156 patients divided into 135 males and 21 females. One hundred and twenty‐five patients were smokers (80.13%), and 118 were men. A recurrence rate of 43.6% (68 patients) was observed in the complete sample. We found a relationship between recurrence and smoking (*p* = 0.002) and between recurrence and male gender (*p* = 0.015). In comparison with the three types of treatment, intercostal drainage had the highest recurrence rate (52.9%), and surgical treatment had the lowest rate (6.8%). In both surgical and conservative treatments, all recurrent cases were male smokers.

**Conclusion:**

In Syria, the high prevalence of smoking, especially among males, significantly increases the risk and recurrence of spontaneous pneumothorax. These findings highlight the need for targeted smoking cessation programs and should inform the selection of treatment strategies for SP patients, particularly those at high risk of recurrence.

## 1 Introduction

Pneumothorax is a case where air is collected in the pleural space between the lungs and the chest wall. It is considered an important medical condition and often an emergency. The reasons for its occurrence are not entirely clear, and it may be spontaneous or caused by medical conditions or trauma. Spontaneous pneumothorax (SP) is classified into primary, where the patient has no history of lung disease, and secondary, which occurs in the presence of existing lung disease. Diagnosis of pneumothorax is based on clinical symptoms and is confirmed by radiography. The estimated incidence of spontaneous pneumothorax is 17–24 per 100,000 in males and 1.0–6.0 per 100,000 in females [1, 2].

One of the main risk factors for SP, which has been linked to several other causes, is smoking. Studies have shown that smoking has pathological effects on the airways, causing widespread bronchiolitis, which increases the risk of occurrence and recurrence of SP [3, 4].

Although no standardized, evidence‐based treatment for pneumothorax is agreed upon by all health institutions, all treatments have the same goal. This goal is to remove air from the pleural space, achieve full lung diffusion, eliminate the risk of it turning into tension pneumothorax, and reduce its other complications and the possibility of recurrence [4, 5].

To the best of our knowledge, no studies have been conducted in our region on spontaneous pneumothorax. Therefore, we conducted a study that included patients with spontaneous pneumothorax who were treated in Al‐Mouwasat and Al‐Assad University Hospitals in Damascus over a period of 5 years. We aimed to determine the distribution of patients according to demographic data in our society, the prevalence of smoking among patients, the treatment methods used, the recurrence rate, along with the impact of smoking on recurrence.

## 2 Methods

### 2.1 Study Design and Settings

This study is a cross‐sectional study that has included data from patients with spontaneous pneumothorax who were admitted to Al‐Assad and Al‐Mouwasat University Hospitals in Damascus, during the period from January 2016 to January 2021, after obtaining the necessary approvals from the Faculty of Medicine and the Scientific Research Council at the University of Damascus.

### 2.2 Study Population, Inclusion, and Exclusion Criteria

The basis for diagnosis was a chest X‐ray following clinical suspicion in patients suffering from chest pain, shortness of breath, and decreased breath sounds on the affected side of the chest. CT scans were only performed in cases of suspected emphysema.

Patients with traumatic, iatrogenic, or hydatid cyst‐related pneumothorax, as well as those under 15 or over 80 years old, were excluded.

The total number of patients with pneumothorax was 395, and after applying the exclusion criteria mentioned above, the study sample size was 156 patients, including 135 males and 21 females.

### 2.3 Sample Size

To determine the appropriate sample size (*n*), we utilized Cochran's Sample Size Formula. The calculations were based on several factors, including a 95% confidence level (represented by *Z* = 1.96), a margin of error (represented by e), and an estimated proportion (*p*) of the population that possesses the attribute of interest of 50% (or 0.5). The value of *q* was calculated as 1 − *p*:

$$n=\frac{z^{2}\mathrm{pq}}{{\mathrm{e}}^{2}}.$$

The required sample size (*n*) for this study, applying the previous formula, is 114.

### 2.4 Variables and Treatments

The study included several variables, including gender, age, height, weight, body mass index (BMI), smoking status, smoking rate estimated by pack‐years (a pack‐year is defined as 20 cigarettes smoked every day for 1 year), date of onset, type of treatment applied, and recurrence. Treatments were classified into three categories: conservative treatment, intercostal drainage (chest tube) with local anesthesia, and surgical treatment (including video‐assisted thoracoscopic surgery and thoracotomy) under general anesthesia. Patients were followed up for 2 years to monitor the recurrence after treatment.

### 2.5 Ethical Approval and Consent to Participate

The authors assert that all procedures contributing to this study comply with the ethical standards of the relevant national and institutional committees on human experimentation and with the Helsinki Declaration of 1975, as revised in 2008. The Ethical Committee approved this study in the Faculty of Medicine in Damascus University, Damascus, Syrian Arab Republic with number (7149). All our methods were carried out following relevant guidelines and regulations. Informed consent was obtained from all the participants included in the study. We explained the purpose of the study to each participant, and it was all voluntary. No names were taken, so we provided anonymous data collection.

### 2.6 Statistical Analysis

Statistics were used to express the numerical variables as Mean and Standard Deviation (SD), and the categorical variables as numbers and percentages. All statistical analyses were performed using SPSS version 22. We used Pearson's Chi‐square test to assess the association between smoking and SP recurrence. A two‐sided significance level of 0.05 was used for all tests. The primary analysis, pre‐specified before data collection, focused on the relationship between smoking and SP recurrence. Subgroup analyses were exploratory and were not pre‐specified.

## 3 Results

This study included 156 patients diagnosed with spontaneous pneumothorax within the included period, divided into 135 males and 21 females.

Table 1 shows the distribution of patients according to sex and smoking status. The average height, weight, and body mass were calculated for each group, and the values of these averages were within the normal range without any significant differences between the groups.

**TABLE 1:** Comparisons of clinical features between smokers and nonsmokers.

Variable	Smokers			Non‐smokers		
	Male	Female	Total	Male	Female	Total
**Count**	118	7	125	17	14	31
Age yr, (SD)	42.52 (16.74)	38.42 (13.02)	42.3 (16.54)	41.94 (22.74)	30.29 (10.81)	36.68 (19)
Height cm, (SD)	174.6 (8)	168 (7.05)	174.18 (8.08)	177.4 (8.83)	160.6 (6.24)	171.22 (11.42)
Weight kg, (SD)	69.02 (16.32)	60.29 (5.68)	68.45 (15.98)	70.47 (12.7)	58.18 (16.98)	65.64 (15.48)
BMI, (SD)	22.66 (5.18)	21.46 (2.83)	22.58 (5.06)	22.46 (4.63)	24.01 (5.48)	23.04 (4.76)

The patients with SP in our study were predominantly male, accounting for 86.5% of the cases. Additionally, a large majority of the patients were smokers, with a prevalence of 80.13% (125/156). In comparison, the prevalence of smoking among females with SP is much lower (33.3%) than among males (87.4%).

In Table 2, the patients were categorized based on their smoking habits, measured in pack‐years. Patients who smoked water pipes (six in total) were placed in a separate column as their pack‐year score could not be calculated. The distribution of patients showed a significant increase in the number of patients as the smoking dose increased, suggesting a correlation between smoking habits and the occurrence of SP. Among the patients, the highest percentage of smokers was in the age group between 40 and 59 years, where out of 49 patients, 43 were smokers (87.7%). On the other hand, patients aged 15–24 years had the lowest prevalence of smoking compared to the other age groups, with 23 out of 34 patients being smokers (67.6%).

**TABLE 2:** Distribution by age and smoking habits.

	Pack. Year						
Age, yr	0	1–19	20–39	40–59	59<	Water pipe	Total
15–24	11	19	1	0	0	3	34
25–39	9	15	8	10	1	1	44
40–59	6	5	21	4	11	2	49
60–80	5	0	7	4	13	0	29
Total	31	39	37	18	25	6	156

In Table 3, the rates of recurrence among patients were compared based on their sex, smoking status, and type of treatment, as patients underwent three different types of treatments. Additionally, 18 patients received intercostal drainage and later underwent surgical treatment after experiencing recurrent pneumothorax.

**TABLE 3:** Recurrence rate by smoking status, sex and type of treatment.

Risk factors	Recurrence *n* = 68	*p*‐Value
Smoking	62 (91.2%)	0.002*
Male	64 (92.7%)	0.015*
Treatment *n* (%)		
Intercostal drainage *n* = 119	63 (52.9%)	
Smoking	57	0.008*
Male	59	0.052
Surgical *n* = 44	3 (6.8%)	
Smoking	3	0.521
Male	3	0.435
Conservative *n* = 11	2 (18.2%)	
Smoking	2	0.087
Male	2	0.461

* = *p*‐value < 0.05

A recurrence rate of 43.6% (68/156) was observed in the complete sample. The recurrence of SP was found to be associated with smoking, and there was a statistically significant difference between smokers and non‐smokers. (*X*²(1, *N* = 156) = 9.241, *p* = 0.002; OR = 4.10, 95% CI: 1.57–10.68).

Among the three treatments, intercostal drainage had the highest rate of recurrence (52.9%). Relapse after this treatment was also found to be associated with smoking, with a significant statistical value of (*X* ^2^(1, *N* = 119) = 7.138, *p* = 0.008). Conservative treatment had a recurrence rate of 18.2% (2 out of 11 patients relapsed), but there was no significant statistical value with smoking (*X* ^2^(1, *N* = 11) = 2.933, *p* = 0.087).

As for the surgical treatment, it had the lowest rate of recurrence (6.8%). Of the 44 patients who were treated surgically, only three patients relapsed, without a significant statistical value associated with smoking (*X* ^2^(1, *N* = 44) = 0.413, *p* = 0.512). In both surgical and conservative treatments, all recurrent cases were male smokers.

## 4 Discussion

In this study, we reviewed the files of patients diagnosed with SP in our university hospitals. The prevalence of smoking was high among the patients, and there was a predominance of males in our sample. Females had a lower smoking rate than males. Smoking was found to have a significant effect on recurrence after treatment in the entire sample. While a significant association was found between smoking and recurrence in intercostal drainage, there was no significant statistical value of smoking on recurrence after surgical or conservative treatment separately.

The high prevalence of smoking found in the patients of our study is in line with previous research linking SP and smoking. This habit was found to increase the risk of SP occurrence by 9 times for women and 22 times for men [3]. Previous study also observed a significant relationship with the smoking dose, which was associated with an increased risk of SP in men who smoked heavily throughout their lives to 12%, compared to the rate in non‐smokers of 1/1000 [3]. These findings are consistent with those of our study in (Table 2).

Our study found that the male‐to‐female ratio was 6.4:1, which is slightly higher than the ratios reported in other studies (2.7:1–3.3:1) [6, 7]. Ayed et al. (2006) also found a high male predominance (male‐to‐female ratio of 20.1:1) in Kuwait, similar to our findings [8]. This male predominance can be attributed to the higher prevalence of smoking in men and at a higher dose [3]. as well as the structural difference between males and females that allows females to expand their airways more than men, which increases bronchial infections in men [9].

We believe that the lower prevalence of smoking among females in our conservative society is the reason for the high male‐to‐female ratio in our study regarding the role of smoking in the occurrence of SP. This is supported by another study conducted in Syria, which found that 56.9% of men versus 17.0% of women smoke cigarettes, and 20.2% of men versus 4.8% of women smoke waterpipes [10]. On the other hand, smoking habits have increased, especially in light of the Syrian crisis, which was confirmed by a study conducted on young people in Syrian society [11].

The recurrence rate of SP in our sample, which was 43.6%, is similar to other studies that reported rates ranging from 13% to 52% [2, 5, 12, 13, 14]. Studies have also indicated that smoking has a significant impact on increasing the recurrence of SP [5, 12, 15], which is consistent with our findings (*p* = 0.002; OR = 4.10, 95% CI: 1.57–10.68), where the association between smoking and recurrence was statistically significant. This is because smoking causes bronchitis, which leads to air trapping in the alveoli, resulting in hyperextension and rupture of the alveoli, as well as other mechanisms that cause interstitial lung diseases and airway diseases [15, 16]. Furthermore, smoking cessation after the first occurrence of pneumothorax was associated with a four‐fold reduction in the risk of recurrence [8, 17].

Our study revealed a statistically significant association between recurrence and male sex. However, our findings differ from a French study that reported a higher rate of recurrence in women compared to men (56% vs. 52%) [7]. This difference is likely due to the high prevalence of smoking among men in our society, as previously mentioned, which plays a major role in increasing the recurrence of pneumothorax among men.

In our sample, the majority of patients underwent intercostal drainage during their first incidence of SP. The recurrence rate after drainage was 52.9%, which is consistent with similar studies reporting recurrence rates ranging between 15% and 50% [18, 19, 20, 21, 22, 23]. The association with smoking was statistically significant (*p* = 0.008), as 90.5% of recurrent cases were smokers. This result is expected due to the mechanism of smoking and its role in increasing the risk of recurrence.

In other studies, the recurrence rate after surgical intervention ranged from 1% to 5% [24, 25], while for both primary and secondary SP, the recurrence rate after conservative treatment was 7.2%–26.7% [26]. Our rates were similar, with a recurrence rate of 6.8% after surgical intervention and 18.2% after conservative treatment. Although there is no significant statistical value associated with smoking, the fact that all recurrences were smokers raises suspicion of smoking's role in recurrence for these two types of treatments. However, another study reported that smokers had better results in terms of not recurring pneumothorax after surgical intervention compared to non‐smokers [27]

The recurrence rate in treatment associated with smoking highlights the importance of educating patients, especially men, about the harmful effects of smoking and its danger to their condition. Referring them to smoking cessation clinics is also necessary. It may be beneficial to consider early therapeutic interventions (like pleurodesis) that prevent recurrence without waiting for it, particularly among heavy‐smoker males.

There are several limitations to this study: (1) It is a retrospective study, and the data source was limited to hospitals affiliated with the university; (2) University hospitals tend to collect complex and high‐risk cases; (3) There is a lack of height and weight data in the files of 23 patients that we were unable to collect; (4) The small sample size limits the reliability of the results. To address these limitations, cohort studies should be conducted that consider the aforementioned limitations, include a larger sample size, and accurately determine the factors affecting SP in our area.

## 5 Conclusion

Among patients with SP in Syria, there is a high prevalence of smoking, with most of them being men. Smoking plays a significant role in increasing the risk of developing SP, and it is a crucial factor in increasing the risk of recurrence. Also, the cases of recurrent were associated with the male sex. Therefore, when treating heavy smoking patients, this must be taken into consideration by applying preventive treatment against recurrence when the SP occurs for the first time and referring patients, especially males, to smoking cessation clinics after the first occurrence of SP.

## Author Contributions

**Muhanad Munzer:** conceptualization, writing – original draft, methodology, writing – review and editing, formal analysis, data curation. **Nafiza Martini:** conceptualization, writing – original draft, methodology, writing – review and editing. **Mhd Mustafa Albitar:** conceptualization, writing – original draft, methodology, writing – review and editing. **Lilas Channiss:** conceptualization, writing – original draft, methodology, writing – review and editing. **Mohammed Martini:** writing – review and editing, writing – original draft. **Hussam Al Bardan:** supervision, writing – review and editing.

## Conflicts of Interest

The authors declare no conflicts of interest.

## Transparency Statement

The lead author, Nafiza Martini, affirms that this manuscript is an honest, accurate, and transparent account of the study being reported; that no important aspects of the study have been omitted; and that any discrepancies from the study as planned (and, if relevant, registered) have been explained.

## Data Availability

The datasets collected and analyzed during the current study are not publicly available. Some restrictions apply to the availability of these data but are available from the corresponding author upon reasonable request.

## References

[1] C. L. McKnight and B. Burns Pneumothorax. StatPearls. Treasure Island (FL) Ineligible Companies. Disclosure: Bracken Burns Declares no Relevant Financial Relationships With Ineligible Companies: StatPearls Publishing Copyright © 2023, StatPearls Publishing LLC.; 2023.

[2] R. J. Hallifax , R. Goldacre , M. J. Landray , N. M. Rahman , and M. J. Goldacre , “Trends in the Incidence and Recurrence of Inpatient‐Treated Spontaneous Pneumothorax, 1968–2016,” Journal of the American Medical Association 320, no. 14 (2018): 1471–1480. 30304427 10.1001/jama.2018.14299 PMC6233798

[3] L. Bense , G. Eklund , and L. G. Wiman , “Smoking and the Increased Risk of Contracting Spontaneous Pneumothorax,” Chest 92, no. 6 (1987): 1009–1012. 3677805 10.1378/chest.92.6.1009

[4] J. M. Tschopp , R. Rami‐Porta , M. Noppen , and P. Astoul , “Management of Spontaneous Pneumothorax: State of the Art,” European Respiratory Journal 28, no. 3 (2006): 637–650. 16946095 10.1183/09031936.06.00014206

[5] O. Bintcliffe and N. Maskell , “Spontaneous Pneumothorax,” BMJ 348 (2014): g2928. 24812003 10.1136/bmj.g2928

[6] D. Gupta , “Epidemiology of Pneumothorax in England,” Thorax 55, no. 8 (2000): 666–671. 10899243 10.1136/thorax.55.8.666 PMC1745823

[7] A. Bobbio , A. Dechartres , S. Bouam , et al., “Epidemiology of Spontaneous Pneumothorax: Gender‐Related Differences,” Thorax 70, no. 7 (2015): 653–658. 25918121 10.1136/thoraxjnl-2014-206577

[8] A. K. Ayed , S. Bazerbashi , M. Ben‐Nakhi , et al., “Risk Factors of Spontaneous Pneumothorax in Kuwait,” Medical Principles and Practice 15, no. 5 (2006): 338–342. 16888390 10.1159/000094266

[9] L. M. Taussig , K. Cota , and W. Kaltenborn , “Different Mechanical Properties of the Lung in Boys and Girls,” The American Review of Respiratory Disease 123, no. 6 (1981): 640–643. 7271061 10.1164/arrd.1981.123.6.640

[10] K. D. Ward , T. Eissenberg , S. Rastam , et al., “The Tobacco Epidemic in Syria,” Tobacco Control 15, no. Suppl 1 (2006): i24–i29. 16723671 10.1136/tc.2005.014860 PMC2563543

[11] A. Kakaje , M. M. Alhalabi , A. Alyousbashi , A. Ghareeb , L. Hamid , and A. B. Al‐Tammemi , “Smoking Habits and the Influence of War on Cigarette and Shisha Smoking in Syria,” PLoS One 16, no. 9 (2021): e0256829. 34473786 10.1371/journal.pone.0256829 PMC8412248

[12] F. Schramel , P. Postmus , and R. Vanderschueren , “Current Aspects of Spontaneous Pneumothorax,” European Respiratory Journal 10, no. 6 (1997): 1372–1379. 9192946 10.1183/09031936.97.10061372

[13] B. V. Udelsman , D. C. Chang , M. Lanuti , D. J. Mathisen , and A. Muniappan , “Risk Factors for Recurrent Spontaneous Pneumothorax: A Population Level Analysis,” The American Journal of Surgery 223, no. 2 (2022): 404–409. 34119331 10.1016/j.amjsurg.2021.05.017

[14] O. J. Bintcliffe , R. J. Hallifax , A. Edey , et al., “Spontaneous Pneumothorax: Time to Rethink Management?,” The Lancet Respiratory Medicine 3, no. 7 (2015): 578–588. 26170077 10.1016/S2213-2600(15)00220-9

[15] Y. L. Cheng , T. W. Huang , C. K. Lin , et al., “The Impact of Smoking in Primary Spontaneous Pneumothorax,” The Journal of Thoracic and Cardiovascular Surgery 138, no. 1 (2009): 192–195. 19577078 10.1016/j.jtcvs.2008.12.019

[16] S. R. Desai , S. M. Ryan , and T. V. Colby , “Smoking‐Related Interstitial Lung Diseases: Histopathological and Imaging Perspectives,” Clinical Radiology 58, no. 4 (2003): 259–268. 12662946 10.1016/s0009-9260(02)00525-1

[17] S. P. Walker , A. C. Bibby , P. Halford , L. Stadon , P. White , and N. A. Maskell , “Recurrence Rates in Primary Spontaneous Pneumothorax: A Systematic Review and Meta‐Analysis,” European Respiratory Journal 52, no. 3 (2018): 1800864. 30002105 10.1183/13993003.00864-2018

[18] M. Noppen , P. Alexander , P. Driesen , H. Slabbynck , and A. Verstraeten , “Manual Aspiration Versus Chest Tube Drainage in First Episodes of Primary Spontaneous Pneumothorax: A Multicenter, Prospective, Randomized Pilot Study,” American Journal of Respiratory and Critical Care Medicine 165, no. 9 (2002): 1240–1244. 11991872 10.1164/rccm.200111-078OC

[19] H. Lippert , O. Lund , S. Blegvad , and H. Larsen , “Independent Risk Factors for Cumulative Recurrence Rate After First Spontaneous Pneumothorax,” European Respiratory Journal 4, no. 3 (1991): 324–331. 1864347

[20] K. K. Ho , M. E. H. Ong , M. S. Koh , E. Wong , and J. Raghuram , “A Randomized Controlled Trial Comparing Minichest Tube and Needle Aspiration in Outpatient Management of Primary Spontaneous Pneumothorax,” The American Journal of Emergency Medicine 29, no. 9 (2011): 1152–1157. 20716475 10.1016/j.ajem.2010.05.017

[21] J. S. Chen , W. K. Chan , K. T. Tsai , et al., “Simple Aspiration and Drainage and Intrapleural Minocycline Pleurodesis Versus Simple Aspiration and Drainage for the Initial Treatment of Primary Spontaneous Pneumothorax: An Open‐Label, Parallel‐Group, Prospective, Randomised, Controlled Trial,” The Lancet 381, no. 9874 (2013): 1277–1282. 10.1016/S0140-6736(12)62170-9 23489754

[22] R. J. Hallifax , A. Yousuf , H. E. Jones , J. P. Corcoran , I. Psallidas , and N. M. Rahman , “Effectiveness of Chemical Pleurodesis in Spontaneous Pneumothorax Recurrence Prevention: A Systematic Review,” Thorax 72, no. 12 (2017): 1121–1131. 27803156 10.1136/thoraxjnl-2015-207967 PMC5738542

[23] S. G. A. Brown , E. L. Ball , S. P. J. Macdonald , C. Wright , and D. McD Taylor , “Spontaneous Pneumothorax; A Multicentre Retrospective Analysis of Emergency Treatment, Complications and Outcomes,” Internal Medicine Journal 44, no. 5 (2014): 450–457. 24612237 10.1111/imj.12398

[24] A. Bille , A. Barker , E. C. Maratos , L. Edmonds , and E. Lim , “Surgical Access Rather Than Method of Pleurodesis (Pleurectomy or Pleural Abrasion) Influences Recurrence Rates for Pneumothorax Surgery: Systematic Review and Meta‐Analysis,” General thoracic and cardiovascular surgery 60, no. 6 (2012): 321–325. 22566267 10.1007/s11748-012-0080-9

[25] P. B. Pagès , J. P. Delpy , P. E. Falcoz , et al., “Videothoracoscopy Versus Thoracotomy for the Treatment of Spontaneous Pneumothorax: A Propensity Score Analysis,” The Annals of Thoracic Surgery 99, no. 1 (2015): 258–263. 25440269 10.1016/j.athoracsur.2014.08.035

[26] H. V. Kwak , K. C. Banks , Y. Y. Hung , et al., “Utilization and Outcomes of Observation for Spontaneous Pneumothorax at an Integrated Health System,” Journal of Surgical Research 288 (2023): 28–37. 36948030 10.1016/j.jss.2023.02.031

[27] H. Uramoto , H. Shimokawa , and F. Tanaka , “What Factors Predict Recurrence of a Spontaneous Pneumothorax?,” Journal of Cardiothoracic Surgery 7 (2012): 112. 23075329 10.1186/1749-8090-7-112 PMC3488480

